# Potential value of ctDNA monitoring in metastatic HR + /HER2 − breast cancer: longitudinal ctDNA analysis in the phase Ib MONALEESASIA trial

**DOI:** 10.1186/s12916-023-03017-z

**Published:** 2023-08-15

**Authors:** Joanne Chiu, Fei Su, Mukta Joshi, Norikazu Masuda, Takashi Ishikawa, Tomoyuki Aruga, Juan Pablo Zarate, Naveen Babbar, O. Alejandro Balbin, Yoon-Sim Yap

**Affiliations:** 1 Queen Mary Hospital, 102 Pok Fu Lam Rd, Pok Fu Lam, Hong Kong; 2 Novartis Pharmaceuticals Corporation, 1 Health Plaza, East Hanover, NJ USA; 3 Novartis Institutes for BioMedical Research, 250 Massachusetts Ave, Cambridge, MA USA; 4 Nagoya University Graduate School of Medicine, Building B, Furocho, Chikusa Ward, Nagoya, Japan; 5 Tokyo Medical University Hospital, 6 Chome-7-1 Nishishinjuku, Shinjuku City, Tokyo Japan; 6 Tokyo Metropolitan Komagome Hospital, 3 Chome-18 Honkomagome, Bunkyo City, Tokyo Japan; 7 Division of Medical Oncology, National Cancer Centre Singapore, 30 Hospital Blvd, Singapore, Singapore

**Keywords:** Ribociclib, MONALEESASIA, Advanced breast cancer, CDK4/6 inhibitor, ctDNA, Longitudinal analysis

## Abstract

**Background:**

There is increasing interest in the use of liquid biopsies, but data on longitudinal analyses of circulating tumor DNA (ctDNA) remain relatively limited. Here, we report a longitudinal ctDNA analysis of MONALEESASIA, a phase Ib trial evaluating the efficacy and safety of ribociclib plus endocrine therapy (ET) in Asian patients with hormone receptor–positive, human epidermal growth factor receptor-2–negative advanced breast cancer.

**Methods:**

MONALEESASIA enrolled premenopausal and postmenopausal Japanese and postmenopausal non-Japanese Asian patients. All patients received ribociclib with ET (letrozole, fulvestrant, or tamoxifen with goserelin). ctDNA was analyzed using a targeted next-generation sequencing panel of 572 cancer-related genes and correlated by best overall response (BOR).

**Results:**

Five hundred seventy-four cell-free DNA samples from 87 patients were tested. The most frequently altered genes at baseline included *PIK3CA* (29%) and *TP53* (22%). Treatment with ribociclib plus ET decreased ctDNA in most patients at the first on-treatment time point, regardless of dose or ET partner. Patients with partial response and stable disease had lower ctDNA at baseline that remained low until data cutoff if no progressive disease occurred. Most patients with progressive disease as the best response had higher ctDNA at baseline that remained high at the end of treatment. For patients with partial response and stable disease with subsequent progression, ctDNA increased towards the end of treatment in most patients, with a median lead time of 83 days (14–309 days). In some patients with BOR of partial response who experienced disease progression later, specific gene alterations and total ctDNA fraction increased; this was sometimes observed concurrently with the development of new lesions without a change in target lesion size. Patients with alterations in *PIK3CA* and *TP53* at baseline had shorter median progression-free survival compared with patients with wild-type *PIK3CA* and *TP53*, 12.7 and 7.3 months vs 19.2 and 19.4 months, respectively (*P* = .016 and *P* = .0001, respectively).

**Conclusions:**

Higher ctDNA levels and *PIK3CA* and *TP53* alterations detected at baseline were associated with inferior outcomes. On-treatment ctDNA levels were associated with different patterns based on BOR. Longitudinal tracking of ctDNA may be useful for monitoring tumor status and detection of alterations with treatment implications.

**Trial registration:**

ClinicalTrials.gov NCT02333370. Registered on January 7, 2015.

**Supplementary Information:**

The online version contains supplementary material available at 10.1186/s12916-023-03017-z.

## Background

The combination of a cyclin-dependent kinase 4/6 inhibitor (CDK4/6i) and endocrine therapy (ET) has become the new standard of care for patients with hormone receptor–positive, human epidermal growth factor receptor-2–negative (HR + /HER2 −) advanced breast cancer (ABC) [1]. All 3 MONALEESA trials, MONALEESA-2, MONALEESA-3, and MONALEESA-7, have reported significant progression-free survival (PFS) and overall survival (OS) benefits with ribociclib plus ET in the intention-to-treat populations [2–7]. The phase Ib MONALEESASIA trial was designed to evaluate the efficacy and safety of ribociclib plus ET in Asian patients with HR + /HER2 − ABC [8].

MONALEESASIA included dose-escalation and dose-expansion phases of ribociclib plus ET (letrozole, fulvestrant, or tamoxifen plus goserelin) in patients with HR + /HER2 − ABC in Japan, Hong Kong, and Singapore [8]. The clinical benefit rate was ≥ 75% in Japanese patients and 66.7% and 87.0% in non-Japanese patients receiving 400 mg and 600 mg of ribociclib plus letrozole, respectively [8]. In Japanese patients, overall response rates (ORR) of 33.3% (ribociclib [400 mg] plus letrozole), 59.1% (ribociclib [300 mg] plus letrozole), 66.7% (ribociclib plus tamoxifen), and 18.8% (ribociclib plus fulvestrant) were observed [8]. In non-Japanese patients receiving 400 mg and 600 mg ribociclib plus letrozole, ORRs of 50.0% and 56.5% were observed, respectively [8]. An exploratory gene-expression analysis on serial biopsy specimens demonstrated decreased expression of a subset of genes of interest (including E2F-responsive genes such as *MYC*, *TYMS*, and cell cycle–related genes) during treatment (cycle 1 day 15 [C1D15]) from baseline [8].

Although tumor biopsy remains the preferred clinical diagnostic method in oncology, liquid biopsy technologies have been increasingly adopted to obtain predictive and prognostic data [9]. Liquid biopsy of bodily fluids is less invasive and allows successive samplings rather than a single, invasive traditional tumor biopsy. Circulating tumor DNA (ctDNA) obtained from cell-free DNA (cfDNA) in liquid biopsy samples contains biomarkers that could be used to predict response to therapy. With the advancement of ctDNA technology, the use of ctDNA as a surrogate biomarker to monitor disease progression and detect minimal residual disease and resistance mechanisms has been explored in clinical trials [10, 11].

However, limited data are available on longitudinal ctDNA analysis of gene alterations following treatment with CDK4/6i for ABC. Most reports have focused on early ctDNA changes in order to correlate ctDNA with response to treatment [12–14]. For example, in a study of patients with HR + /HER2 − ABC treated with palbociclib plus fulvestrant, changes in ctDNA levels from baseline to C1D15 were predictive of outcomes [15]. Similarly, ctDNA analysis of the real-world POLARIS study determined that early increases (C2D1) in ctDNA levels of patients with HR + /HER2 − ABC correlated with disease progression in patients receiving palbociclib [16]. Although a recent study in 33 patients receiving palliative CDK4/6i reported that serial ctDNA monitoring with a 52-gene assay predicted disease progression with a 2- to 3-month lead time, [17] few studies in ABC have tracked and reported ctDNA dynamics with large gene panels and concurrent measurements using Response Evaluation Criteria in Solid Tumors version 1.1 (RECIST 1.1). Here we present results of a longitudinal ctDNA analysis from MONALEESASIA and correlate changes in the ctDNA fraction, as well as specific gene alterations, with therapeutic response.

## Methods

### Study design and participants

The MONALEESASIA (NCT02333370) study design was previously published [8]. In brief, from February 4, 2015, to March 27, 2017, 88 patients with HR + /HER2 − ABC were enrolled into 2 groups: non-Japanese and Japanese patients. All patients were postmenopausal, except for Japanese patients who were peri- or premenopausal. Patients had histologically and/or cytologically confirmed HR + /HER2 − breast cancer with adequate bone marrow and organ function.

Both Japanese and non-Japanese patients underwent dose-escalation and dose-expansion phases. In the dose-escalation phase, non-Japanese patients received ribociclib (400 mg or 600 mg on a 3-weeks-on/1-week-off schedule) plus letrozole (2.5 mg daily), and Japanese patients received ribociclib (300 mg or 400 mg on a 3-weeks-on/1-week-off schedule) plus letrozole (2.5 mg daily). In the dose-expansion phase, non-Japanese patients received ribociclib (600 mg on a 3-weeks-on/1-week-off schedule) plus letrozole (2.5 mg daily). Dose reduction of ribociclib to a minimum of 200 mg was allowed in both groups [18]. Japanese postmenopausal patients received ribociclib (300 mg on a 3-weeks-on/1-week-off schedule) plus ET: letrozole (2.5 mg daily) or fulvestrant (500 mg on D1 and D15 of C1, then every 28 days). Japanese peri- and premenopausal patients received tamoxifen (20 mg daily with goserelin [3.6 mg every 28 days]) in the dose-expansion phase. Each cycle length was 28 days.

Key exclusion criteria included prior CDK4/6i use, major surgery ≤ 14 days prior to start of study treatment, and a history of cardiac dysfunction or disease. No prior ET or chemotherapy in the advanced setting was permitted, except for in Japanese patients, for whom 1 prior ET for ABC was permitted in the group receiving ribociclib plus fulvestrant.

The primary end points were the maximum tolerated dose and/or recommended Phase 2 dose in the dose-escalation phase and the safety and tolerability of ribociclib plus ET in the dose-expansion phase [8]. Secondary end points were safety and tolerability in the dose-escalation phase and antitumor activity and pharmacokinetics in the dose-escalation and -expansion phases.

Best overall response (BOR) of individual patients was classified as complete response (CR), partial response (PR), stable disease (SD), or progressive disease (PD) according to RECIST 1.1.

### Sample collection and ctDNA analysis

At baseline (C1D1), on treatment (C5D1 and every subsequent 3 cycles), and at the end of treatment (EOT), blood samples were collected in K2-ethylenediaminetetraacetic acid (EDTA)–coated collection tubes and processed within 30 min of collection. ctDNA was extracted from approximately 4 mL of plasma with the QIAamp Circulating Nucleic Acid Kit (Qiagen, Hilden, Germany) per the manufacturer’s instructions and constructed into sequencing libraries with end repair, A-tailing, polymerase chain reaction (PCR) amplification using the TruSeq Nano Library Preparation Kit (Illumina, San Diego, CA). Samples with low ctDNA concentrations were flagged and eliminated from further analysis at 3 steps: ability to make a library compatible with capture (> 200 ng), captured library compatible with sequencing (2 nM), and unique target coverage > 300x. The constructed libraries were sequenced using a targeted panel of 572 cancer-relevant genes (Additional file 1: Panel of Genes Used for cfDNA Analysis) on an Illumina HiSeq instrument to achieve a mean unique coverage of at least 1000 × of pair-end, 100-base-pair reads. Assay performance was assessed in terms of false positives and false negatives down to 1% limit of detection.

Single-nucleotide variants were identified using Mutect (Broad Institute, Cambridge, MA). Indels were identified using Pindel (EMBL Outstation European Bioinformatics Institute, Cambridge, UK) [19, 20]. A position-specific error rate was calculated based on the sequencing of plasma from 24 healthy controls, and mutations were retained only if they had support significantly greater than the position-specific error rate. PureCN (Novartis Institutes for BioMedical Research, Cambridge, MA) was used to call copy number alterations while accounting for ploidy and purity [19]. PureCN purity estimation has been extensively tested [21, 22]. For plasma samples, PureCN sample purity estimation is equivalent to the ctDNA fraction. ctDNA fraction estimation was further refined using an internally developed machine-learning extreme gradient boosting algorithm trained to correct the ctDNA fraction estimation in samples with < 0.03 ctDNA fraction or with spurious single-nucleotide variant calls. Patients with a ctDNA fraction > 0 were considered to have detectable ctDNA, and patients with a ctDNA fraction of 0 were defined as ctDNA undetectable. Copy number amplifications were defined as ≥ 6 and ≥ 7 copies for focal and nonfocal events, respectively, and deletions were defined as 0 copies. Nonframeshift mutations were defined as insertions or deletions that does not cause frameshift. The lowest level of detection was 0.5% irrespective of BOR. Germline mutations and artifacts were filtered out using publicly available databases dbSNP (National Center for Biotechnology Information; Bethesda, MD) and ExAC (Broad Institute) and an internal database (Novartis Institutes for Biomedical Research) of normal ctDNA samples from healthy individuals without cancer. Variants with a mutant allele fraction of < 1% were filtered out unless the variant was a hotspot reported in the COSMIC database (Wellcome Sanger Institute, Hinxton, UK).

Mutations and indels with an allelic fraction of > 40% and no presence in the COSMIC database were also removed because matched normal DNA was not sequenced. Synonymous mutations and indels were removed. Tumor mutational burden calculated from plasma samples (bTMB) was estimated using callMutationBurden in PureCN after removing common and private germline mutations. All comparisons between mutated vs nonmutated (e.g., *TP53* mutated vs nonmutated) were performed on patients with detectable ctDNA.

Lead time to progression was calculated as the difference between a patient’s progression-free survival time (days from baseline) and the first time point between C5D1 and EOT at which the ctDNA fraction was greater than 0. Furthermore, we required that the ctDNA fraction was greater than 0 in at least two consecutive visits. We reason that including only patients that clinically progressed and satisfied the above requirements regarding consecutively confirmed ctDNA fractions greater than 0 would reduce errors due to mutation artifacts that confound ctDNA fraction estimation algorithms.

### Statistical analysis

All statistical and computational analyses were performed in R 4.1.0 (R Foundation, Indianapolis, IN). *t*-tests were used to compare median ctDNA fraction levels across different patient groups by BOR or PFS groups (PFS < 6 months, 6–12 months, 12–24 months, and > 24 months). A Cox proportional-hazards model was used to evaluate differences in PFS in the MONALEESASIA cohorts by ctDNA detectable vs undetectable status and mutation status in *PIK3CA*, *TP53*. Wilcoxon tests were used to calculate the significance of box plots. Oncoprints showing the most frequently mutated genes at baseline and EOT were generated using ComplexHeatmap version 2.8.0 (German Cancer Research Center, Heidelberg, Germany).

## Results

### Patient characteristics and sample collection

Eighty-seven patients from MONALEESASIA were included in this analysis (one enrolled patient withdrew consent; see Additional file 2: Supplementary Table 1). This study included patients from the dose-escalation and dose-expansion phases. A total of 574 ctDNA samples were collected and tested. All 87 patients had baseline ctDNA samples. The median number of on-treatment sample collection time points ranged from 3.5 to 16. At C5D1, samples from 76 of 87 patients (87.4%) were analyzed, and samples from 57 of 87 patients (65.5%) were analyzed at EOT. At the data cutoff for this biomarker analysis (February 15, 2021), 14 patients were still receiving study treatment and had not reached EOT. Samples tested but not included in this analysis were excluded due to quality control failure.

### ctDNA fraction by BOR and PFS

**Fig. 1:**
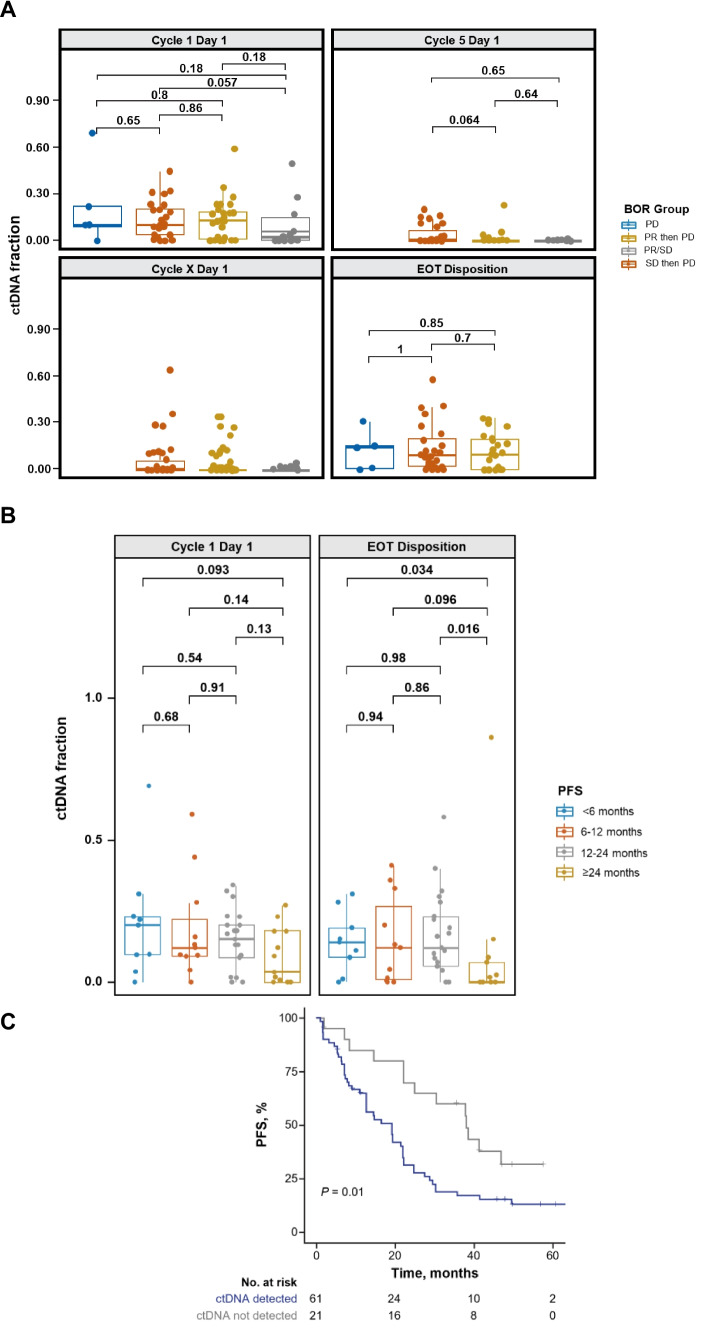
ctDNA fraction by **A** best overall response, **B** PFS, **C** PFS by baseline ctDNA levels. Abbreviations: AE, adverse event; C1D1, cycle 1 day 1; ET, endocrine therapy; NA, not available; PD, progressive disease; PR, partial response; SD, stable disease; WOC, withdrawal of consent

At the data cutoff for this analysis, 14 patients had PR or SD and disease that had not progressed, 7 patients had PD as the best response, 58 patients had PR or SD and then PD, and no patients had CR (Table 1). Comparison of the ctDNA fraction at baseline for these patient groups showed numerical differences in median ctDNA fraction between patients with PR/SD at the time of data cut-off and all others, although the differences were not statistically significant based on a Wilcoxon test (*P* ≥ 0.29; Fig. 1A). However, no differences were observed in ctDNA fraction by patient response during treatment or at EOT for these groups (Wilcoxon *P* ≥ 0.77; the PR/SD group was not included as they did not have EOT). During treatment, the median ctDNA fraction showed no difference between patients with PR/SD and those with PR initially then whose disease progressed (*P* = 0.64 for C5D1) and PR/SD vs those with SD whose disease progressed (*P* = 0.65 for C5D1). To gain a detailed view of the duration of response, we grouped patients by PFS into 4 groups: < 6 months, 6 to 12 months, 12 to 24 months, and > 24 months (Fig. 1B). At baseline, patients with PFS > 24 months showed numerically lower ctDNA fraction (median ctDNA, 0.036) than all other groups (< 6 months, 0.200; 6–12 months, 0.120; 12–24 months, 0.150), although the differences were not statistically significant due to small sample sizes (*P* = 0.093 vs < 6 months, *P* = 0.14 vs 6–12 months, and *P* = 0.13 vs 12–24 months, respectively). A significant difference was noted at EOT between the median ctDNA fraction of patients with PFS ≥ 24 months (med-ctDNA frac, 0.000) compared with patients with PFS < 6 months (med-ctDNA frac, 0.140; *P* = 0.034) or 12 to 24 months (med-ctDNA frac, 0.120; *P* = 0.016). For patients with PFS 6–12 months, a numeric difference was observed (med-ctDNA frac, 0.120), although this difference was not statistically significant vs patients with PFS ≥ 24 months (*P* = 0.096). More generally, patients with no detectable ctDNA at baseline had a PFS almost twice that observed in patients with detectable ctDNA at baseline: 38.0 months vs 19.2 months, respectively (hazard ratio [HR], 0.45; 95% CI, 0.24–0.84; *P* = 0.01; Fig. 1C).

**Table 1:** Patient and tumor characteristics of the biomarker population according to the latest disposition

	**Total biomarker population** ***N* = 87**	**Best overall response and latest status**^**a**^			
		**PD** ***n* = 7**	**PR/SD, then PD** ***n* = 58**	**PR/SD** ***n* = 14**	**AE/WOC**^**b**^ ***n* = 8**
**Age, median (range), years**	NA	54 (41–72)	56 (34–83)	62 (44–80)	66 (53–78)
**Region**					
Japan	58	2	42	9	5
Non-Japan	29	5	16	5	3
**Site of metastases**					
Bone	49	3	37	6	3
Lung/liver	49	6	30	8	5
**Number of metastatic sites**					
< 3	50	3	37	7	3
≥ 3	37	4	21	7	5
**Disease status**^**c**^					
De novo	38	1	28	7	2
**Non-de novo**					
≤ 12 months	1	0	1	0	0
> 12 to ≤ 24 months	4	2	2	0	0
> 24 months	44	4	27	7	6
**ET partner**					
Letrozole	56	5	33	11	7
Tamoxifen	15	0	12	2	1
Fulvestrant	16	2	13	1	0

*Abbreviations*: *AE* adverse event, *C1D1* cycle 1 day 1, *ET* endocrine therapy, *NA* not available, *PD* progressive disease, *PR* partial response, *SD* stable disease, *WOC* withdrawal of consent

^a^PR/SD then PD: patients that had stable disease or partial response who eventually progressed; PR/SD: patients that had stable disease or partial response who did not progress at the study cutoff; AE/WOC: patients that dropped from the study due to adverse event or withdrawal of consent

^b^Responses were not evaluable

^c^For letrozole and tamoxifen combinations, patients who received (neo)adjuvant therapy for breast cancer were eligible. If the prior (neo)adjuvant therapy included letrozole or anastrozole, patients were required to be disease free between > 12 months from completion of treatment until C1D1. For the fulvestrant combination, in patients who received more than 1 line of ET for metastatic disease (i.e., antiestrogen or aromatase inhibitor), documented evidence of progression in the metastatic setting ≤ 12 months from completion of (neo)adjuvant ET was required

### Longitudinal analysis of ctDNA for disease monitoring and acquired resistance to treatment

**Fig. 2:**
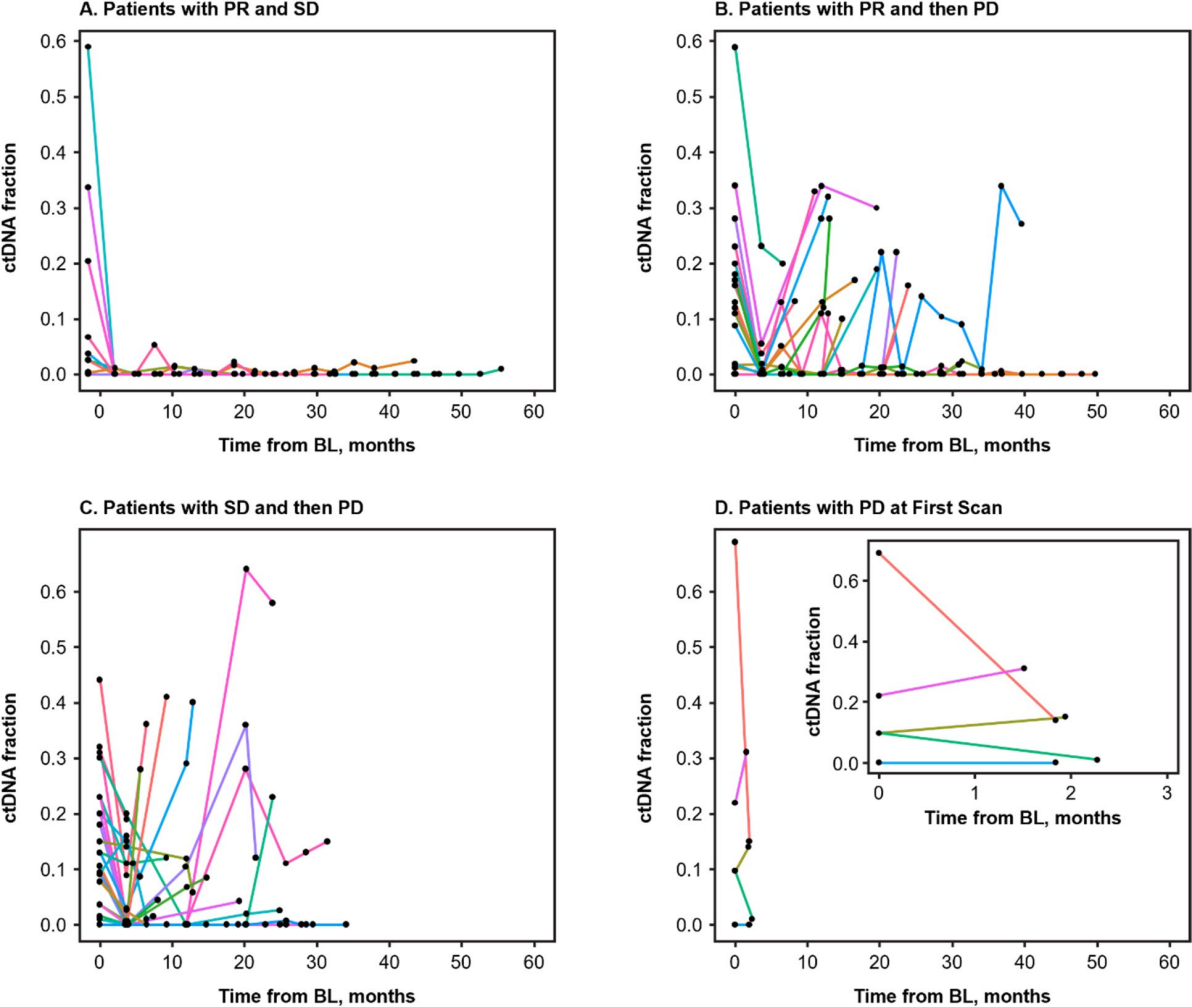
Longitudinal trajectories of ctDNA fraction in patients with evaluable response. Excluded 8 patients who discontinued treatment due to adverse events. Abbreviations: BL, baseline; ctDNA, circulating tumor DNA; PD, progressive disease; PR, partial response; SD, stable disease

**Fig. 3:**
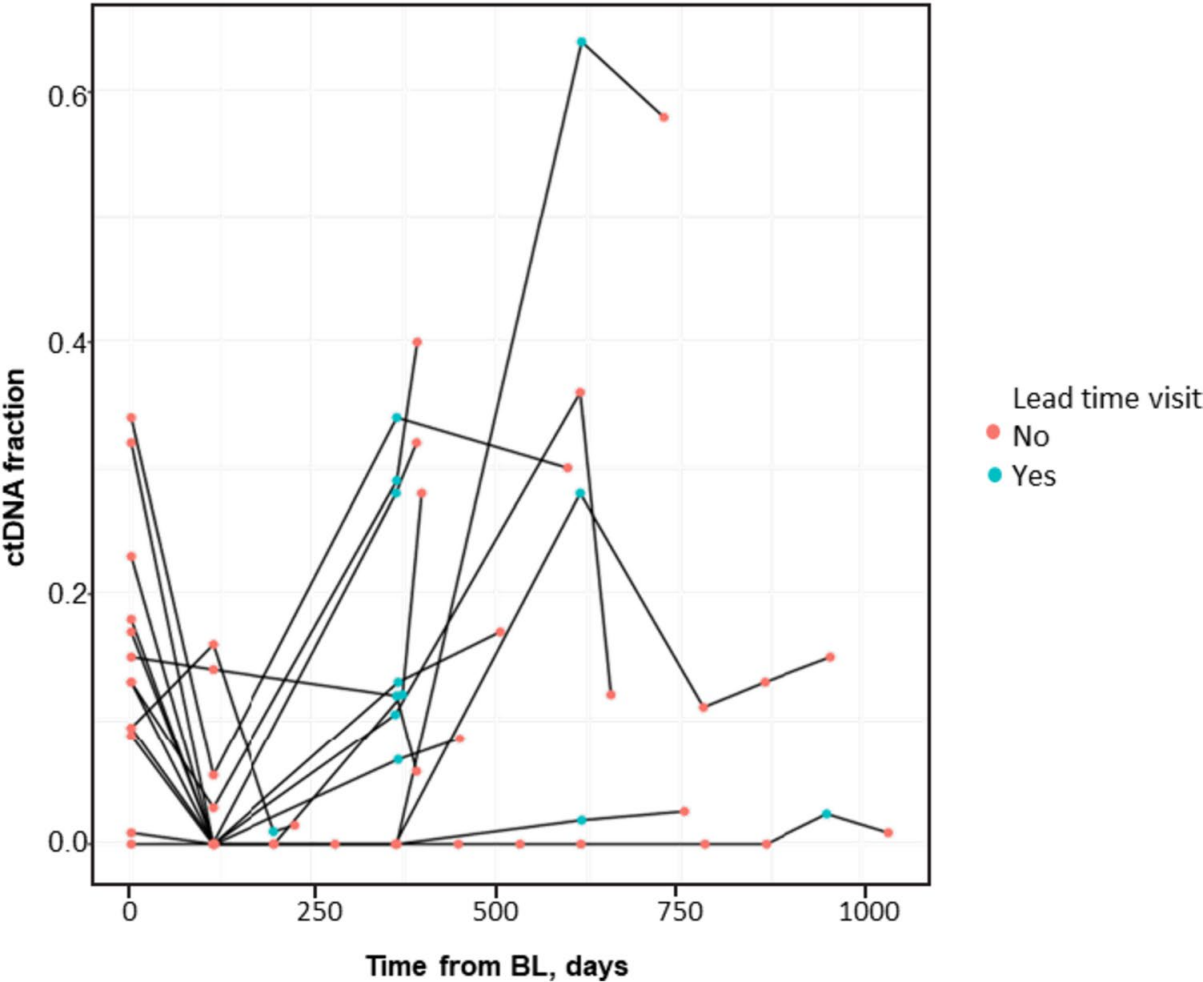
Lead time in detecting PD. Included all patients that clinically progressed and have ctDNA > 0 in 2 consecutive visits. Abbreviations: BL, baseline; ctDNA, circulating tumor DNA. Yes: time point used for calculation of lead time. No: all other timepoints

To track ctDNA fraction from baseline to EOT, a longitudinal analysis of ctDNA levels in individual patients was performed that grouped patients by BOR into 4 categories, as described in the previous section. Patients who had radiological progression at the first scan showed dynamic changes in ctDNA between C1D1 and EOT (Fig. 2D). In patients with PR and SD maintained until the data cutoff (the PR/SD group), ctDNA levels were numerically lower at baseline; notably, these patients maintained low or undetectable ctDNA throughout all on-treatment time points with ribociclib plus ET (Fig. 2A). In contrast, in patients with initial PR or SD who then progressed, ctDNA levels were dynamic throughout treatment with ribociclib plus ET, decreasing at C5D1 and increasing prior to or at PD (Fig. 2B, C). We calculated the duration of ctDNA detection lead time to radiological progression, defined as the time difference between a patient’s PFS (measured in days from baseline) and the first time at which ctDNA fraction was > 0 between C5D1 and EOT. For a given patient to be included in this calculation, we required that their ctDNA fraction be > 0 at least two consecutive visits in order to reduce potential errors due to mutation artifacts that confound ctDNA fraction estimation algorithms (see the “Methods” section). On the basis of this approach, we determined that the median lead time in detecting PD was 83 days, with a range of 14 to 309 days, in 13 out of 58 evaluable patients with initial PR or SD whose disease progressed (Fig. 3).

**Fig. 4:**
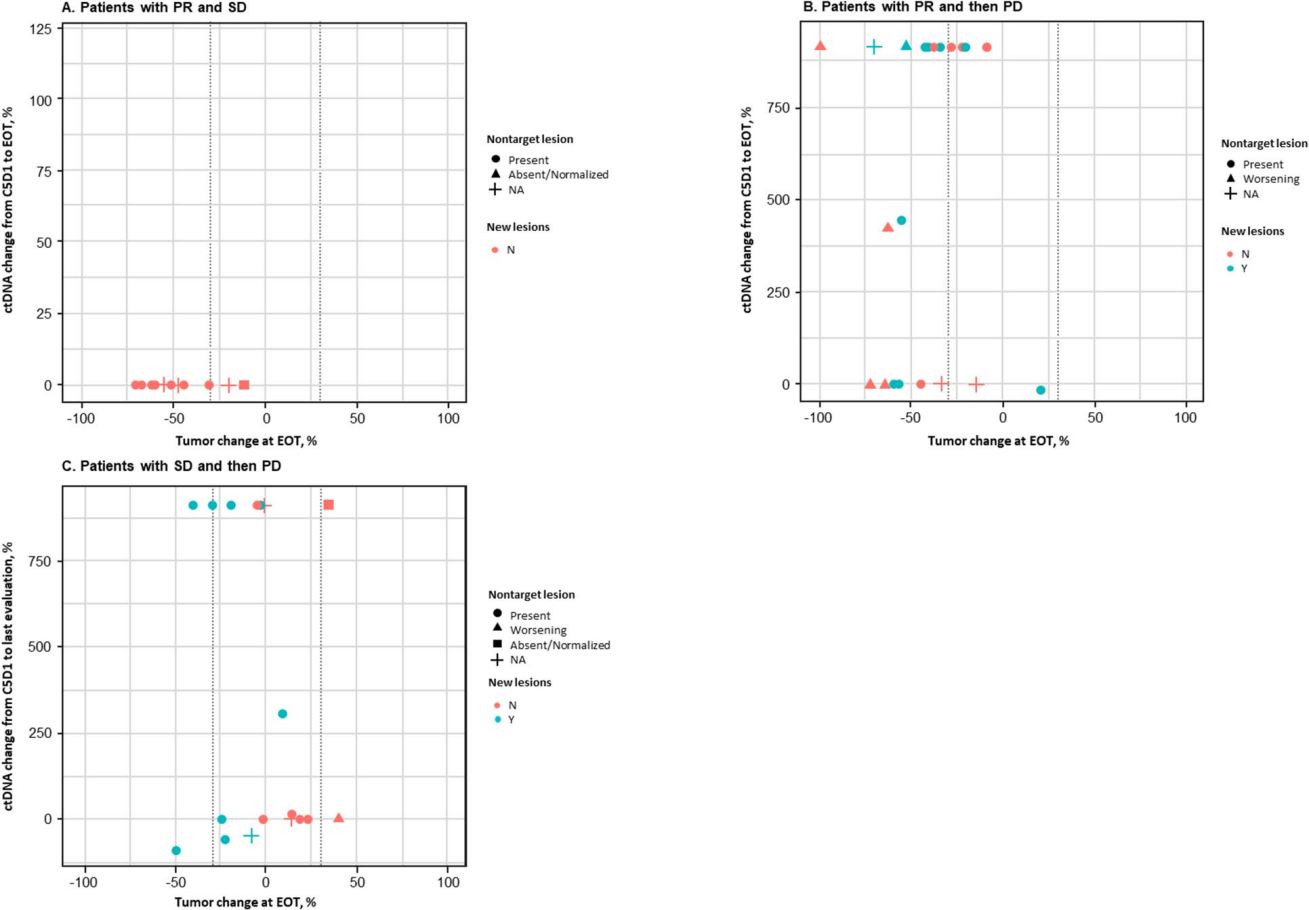
Longitudinal analyses of ctDNA fraction change and correlation with change in tumor diameter by response. Abbreviations: BL, baseline; ctDNA, circulating tumor DNA; NA, not available; PD, progressive disease; PR, partial response; SD, stable disease

The correlation between ctDNA levels and tumor volume with treatment is still poorly understood and can be especially complex for different genetic alterations. Therefore, a longitudinal analysis of the percentage of ctDNA fraction change and its correlation with the percentage of change in tumor diameter was performed for each response group (Fig. 4; Additional file 2: Supplementary Table 2). In the group of patients with PR and SD maintained until the data cut-off, ctDNA levels were low or undetectable and remained so throughout all on-treatment time points, which was consistent with radiographical data, and no new lesions were detected radiologically (Fig. 4A). In the initial PR group whose disease later progressed (Fig. 4B), 12 of 20 patients (60%) had a percentage of ctDNA change > 0 between C5D1 and EOT, which indicated progression; this result was consistent with radiological imaging. Importantly, in 13 of 20 patients (65%), radiological evidence consisted of new lesions or worsening nontarget lesions. In the SD group whose disease then progressed (Fig. 4C), 9 of 17 patients (53%) had an increase in ctDNA between C5D1 and EOT that indicated progression consistent with radiological imaging. Similarly, in 9 of 17 of these patients (53%), the appearance of a new lesion or worsening of nontarget lesions was the major reason for progression. Taken together, for 21 of 37 patients (57%) who showed an initial response and then had disease progression, an increase in ctDNA during treatment was consistent with radiological evidence of progression. Importantly, radiological progression was primarily due to new lesions or worsening nontarget lesions, as opposed to any increase in diameters of target lesions. Of the remaining 16 patients, 6 had nondetectable ctDNA at baseline with our assay.

**Fig. 5:**
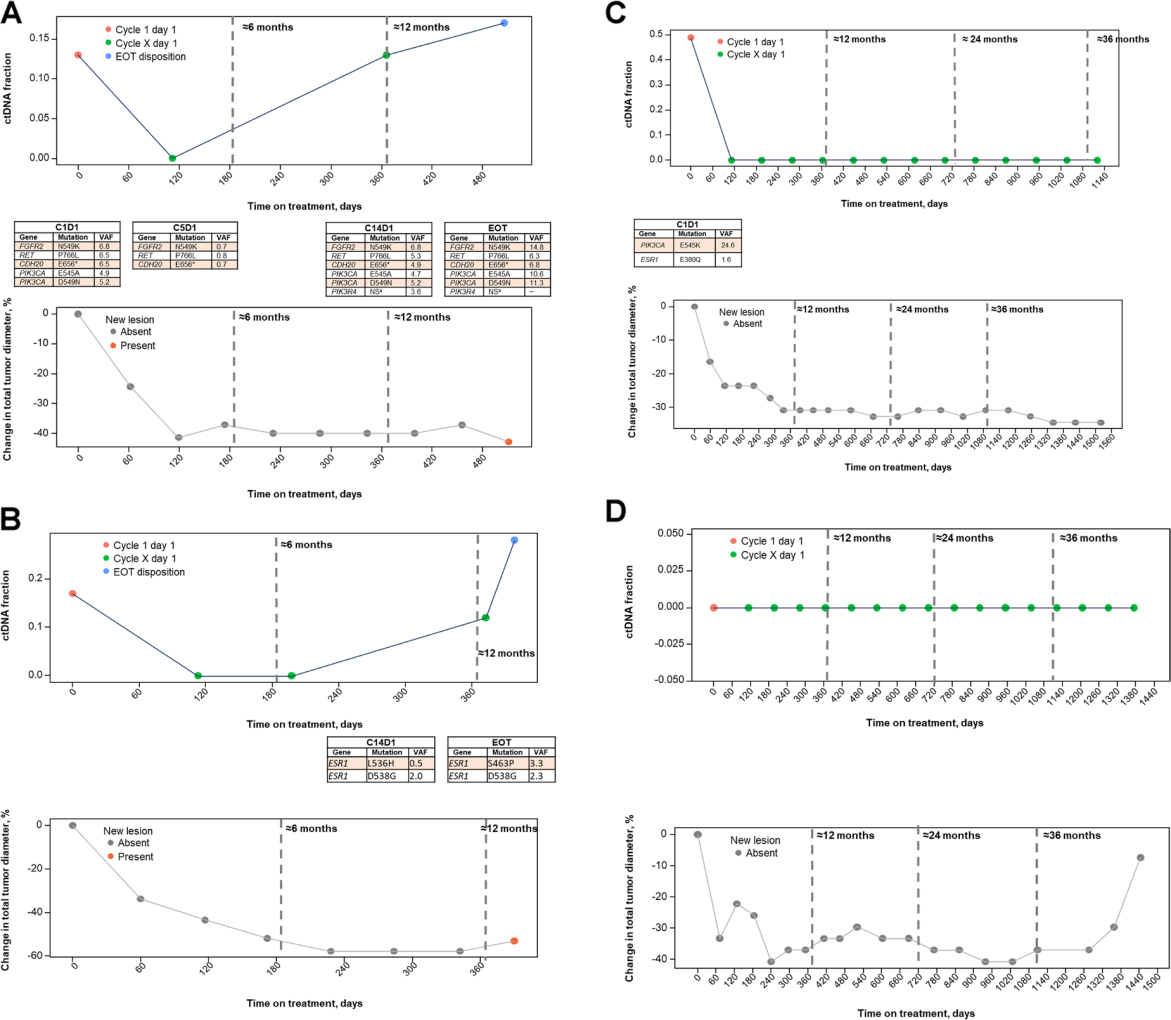
Case studies. **A** Patient with BOR of PR treated with ribociclib 600 mg plus letrozole. At C5D1 (≈120 days), the variant allele fraction (VAF) of these mutations decreased compared with baseline for *FGFR2*, *RET*, and *CDH20*, and VAF levels of *PIK3CA* mutations decreased to below the detection limit. VAF levels of *FGFR2* and *PIK3CA* mutations at EOT were higher than those at C1D1, suggesting that these mutations may be drivers of resistance to treatment. **B** Patient with BOR of PR treated with ribociclib (400 mg) plus letrozole. At C14D1 (≈360 days), this patient acquired 2 *ESR1* mutations (L536H and D538G) with VAF levels of 0.5 and 2.0, respectively. At EOT, the *ESR1* mutation D538G was still present, and an *ESR1* S463P mutation was also detected with VAF levels of 2.3 and 3.3, respectively. **C** Patient with BOR of PR treated with ribociclib (300 mg) plus letrozole. **D** Patient with BOR of PR treated with ribociclib (600 mg) plus letrozole. Abbreviations: AA, amino acid; BOR, best overall response; C1D1, cycle 1 day 1; ctDNA, circulating tumor DNA; EOT, end of treatment; PR, partial response; VAF, variant allele fraction. ^a^ NS indicates a splice site alteration (no AA change), and this alteration may be lost or undetectable at EOT

A more detailed longitudinal analysis of ctDNA change and its correlation with tumor diameter for individual patients was performed (Fig. 5). Figure 5 describes the 4 representative patterns of patients with PR as BOR. The first patient (Fig. 5A) presented with multiple mutations at baseline that were maintained at EOT: 2 *PIK3CA* mutations (E545A, D549N) and 1 mutation each in *FGFR2* (N549K), *RET* (P766L), and *CDH20* (E656*). A new mutation (*PIK3R4*, NS) was acquired at C14D1 (i.e., at ≈360 days). In this patient, ctDNA levels increased at EOT and correlated with progression due to a new lesion, although the size of the targeted tumor did not increase. In a second patient, no gene alterations were detected at baseline (Fig. 5B). However, at C14D1 (≈360 days), this patient had acquired 2 *ESR1* mutations (L536H and D538G). At EOT, the *ESR1* mutation D538G was still present, and an *ESR1* S463P mutation was also detected. A sustained reduction in tumor size was observed in this patient. The aforementioned cases give insight into instances where ctDNA may help detect disease progression before it is detectable by radiological methods. Similar findings of acquired *ESR1* mutations were identified on treatment and at EOT in other patients as well (Additional file 2: Supplementary Fig. 1). A third patient had detectable *PIK3CA* and *ESR1* mutations at baseline that became undetectable in all subsequent visits during the 36 months of treatment, and sustained tumor-size reduction was observed throughout treatment (Fig. 5C). In a fourth patient, no ctDNA was detected at baseline, during treatment, or at EOT; however, the size of the target lesion had increased at EOT, resulting in progression (Fig. 5D).

### Gene alterations at baseline

**Fig. 6:**
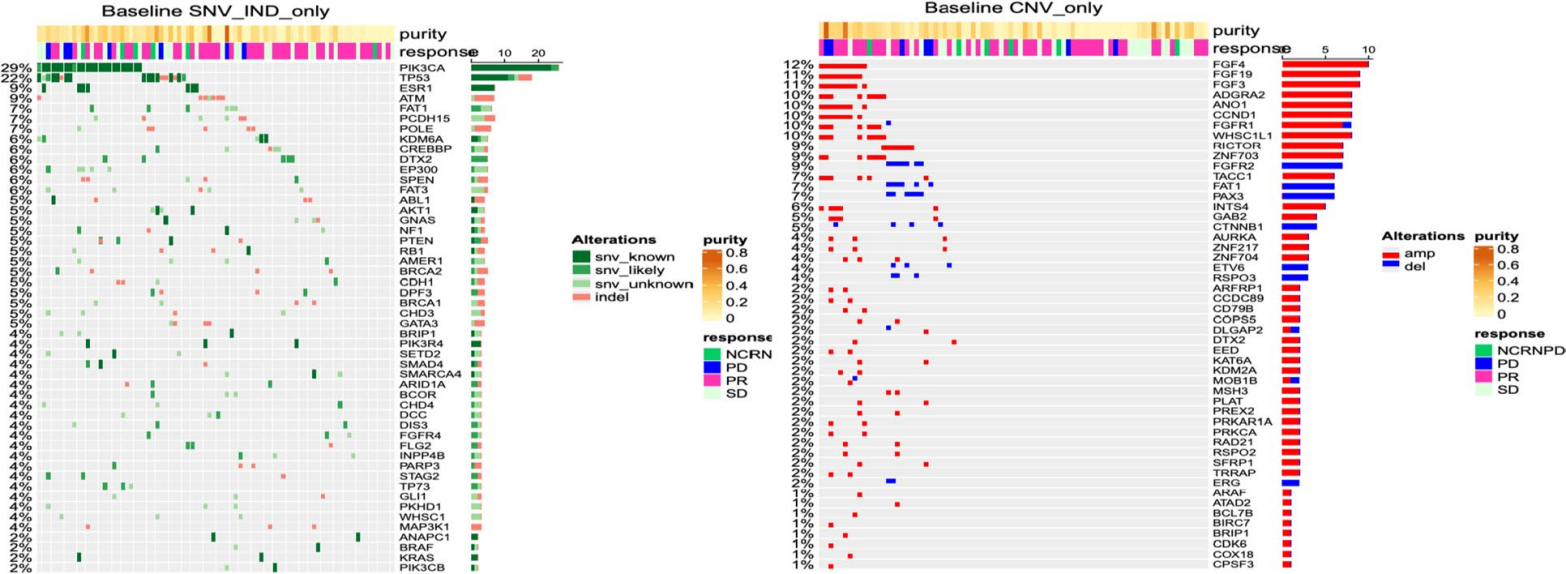
Baseline gene alteration frequency oncoprints: **A** single-nucleotide variants and indels, **B** amplifications and deletions. Abbreviations: amp, amplification; CNV, copy number variant; del, deletion; IND, indel; NCRNPD, noncomplete response or nonprogressive disease; PD, progressive disease; PR, partial response; SD, stable disease; SNV, single-nucleotide variant

Gene alteration frequencies were assessed at baseline, and *PIK3CA* (29%) and *TP53* (22%) were the most frequently altered genes (Fig. 6). Other altered genes of interest included *ESR1* (9%), *NF1* (5%), *PTEN* (5%), and *AKT1* (5%). The most frequently amplified genes were *FGF4* (12%), *FGF19* (11%), *FGF3* (11%), and *CCND1* (10%). These 4 genes are usually coamplified because they are located together on chromosome 11q13; similarly, *FGFR1* (10%), *WHSC1L1* (10%), and ZNF703 (9%), located on chromosome 8p11.23, are usually coamplified (Fig. 6).

**Fig. 7:**
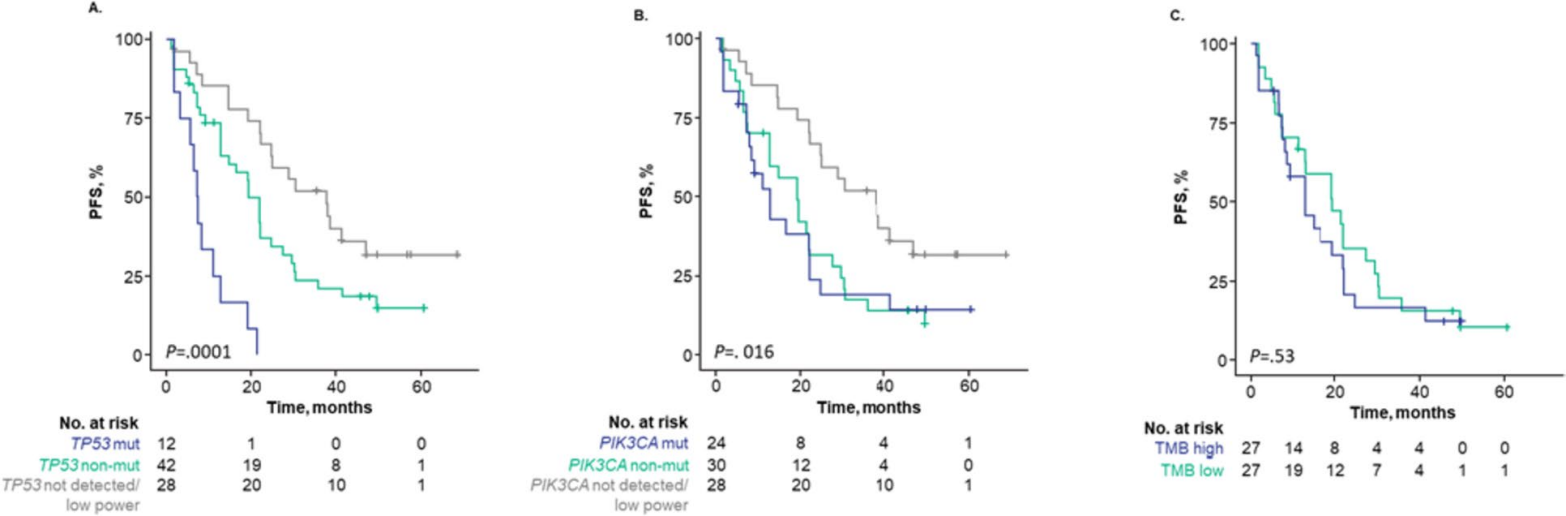
Progression-free survival for ribociclib plus ET in patients with or without mutations. **A** *TP53.* **B** *PIK3CA.* **C** High versus low TMB. Analysis by TMB (high versus low) only included patients with detectable ctDNA. High TMB was defined as > 10 mutations/Mb, and low was defined as ≤ 10 mutations/Mb. Abbreviations: ET, endocrine therapy; mut, mutated; nonmut, nonmutated; PFS, progression-free survival; TMB, tumor mutational burden

*TP53* and *PIK3CA* had the highest mutation frequencies in this analysis; therefore, the PFS benefit of ribociclib plus ET was assessed per baseline mutation status of each gene. Patients with detectable ctDNA and *TP53* or *PIK3CA* mutations at baseline experienced a shorter median PFS (Fig. 7A, B) than patients with detectable ctDNA without alterations in those genes. Median PFS of patients with *TP53* mutations was significantly shorter than those without *TP53* mutations (7.3 months vs 19.4 months; HR, 0.26; 95% CI, 0.12–0.53; *P* < 0.001; Fig. 7A). Median PFS of patients with *PIK3CA* mutations was 12.7 months vs 19.2 months for those without *PIK3CA* mutations; however, the difference was not statistically significant (HR, 0.93; 95% CI, 0.52–1.69; *P* = 0.016; Fig. 7B). Patients with undetectable ctDNA experienced a median PFS of 37.8 months (Fig. 7B). These results should be interpreted with caution due to the small number of patients with select mutations. In patients with detectable ctDNA, the median PFS of patients with high bTMB (> 10 mutations per megabase [mut/Mb]) was 12.7 months vs 19.4 months for those with low bTMB (< 10 mut/Mb); this numerically shorter PFS difference was not statistically significant (HR, 0.83; 95% CI, 0.46–1.5; *P* = 0.53; Fig. 7C). Importantly, in the analyses of bTMB, *TP53*, and *PIK3CA*, which are associated with worst prognosis, were performed only on patients with detectable ctDNA (Fig. 1C). Therefore, in this particular cohort, neither bTMB nor *PIK3CA* status seems to provide an additional prognostic effect beyond ctDNA fraction.

## Discussion

In this longitudinal ctDNA analysis of MONALEESASIA, the most frequently altered genes at baseline were *PIK3CA* and *TP53*, a result similar to ctDNA analysis findings in pooled data from MONALEESA-2, MONALEESA-3, and MONALEESA-7. Patients with alterations in these genes experienced a shorter median PFS compared with patients with wild-type versions [23]. Ribociclib plus ET induced reduction of ctDNA levels in patients who had PR or SD at the first on-treatment sample-collection time point (C5D1), regardless of ribociclib starting dose or ET partner. Patients responding to treatment (i.e., patients with PR and SD as BOR) had lower ctDNA levels at baseline compared with those whose disease progressed (had PD as BOR). The lead time in detecting PD by radiological methods was 14 to 309 days (median, 83 days), consistent with a report using ctDNA to monitor disease progression in patients treated with palbociclib [17]. Generally, our results show that, in 21 of 37 patients (57%) who had an initial response and then progressed (PR then PD; SD then PD), an increase of ctDNA during treatment was consistent with radiological progression (Fig. 4; Supplementary Table 2). Importantly, the radiological progression was primarily due to new lesions or worsening nontarget lesions, as opposed to increases in diameter of target lesions. This finding is consistent with the idea that ctDNA molecular progression is a broader marker that reflects systemic tumor growth beyond the increased diameter of target lesions. Furthermore, more sensitive assays could improve the measurement of ctDNA dynamics in the remaining 16 patients with PD (of 37 patients). Of these 16 patients, 6 had nondetectable ctDNA at baseline (4 had PR then SD; 2 had SD then PD), and it is possible that more sensitive assays would be useful in this ctDNA-negative patient population. Congruently with findings from other studies, [24, 25] we observed a numerically shorter PFS in patients with high bTMB (10 mut/Mb) vs low bTMB (≤ 10 mut/Mb), but the difference was not statistically significant. Although high bTMB is proposed as a potential biomarker for more aggressive tumors, previous analyses included patients with both detectable and undetectable ctDNA, likely confounding their analyses. Our results from patients with detectable ctDNA at baseline indicate that bTMB status might not provide additional prognostic value beyond ctDNA fraction level.

In this analysis, *PIK3CA* mutations, which are associated with poor outcomes and resistance to chemotherapy, were detected at baseline, had dynamic levels of variant allele fraction (VAF) throughout treatment with ribociclib plus ET regardless of BOR, and were associated with a numerically shorter PFS (Fig. 5A, C) [26]. Based on the results of SOLAR-1, alpelisib, a first-in-class PI3Kα inhibitor, has been approved in combination with fulvestrant for use in patients with HR + /HER2 − ABC with *PIK3CA*-mutated tumors who have progressed on first-line CDK4/6i treatment [27, 28]. Identification of *PIK3CA* mutations early in treatment would permit proactive planning for treatment with alpelisib plus fulvestrant prior to progression on first-line CDK4/6i treatment. In this study, acquired *ESR1* mutations were identified in some patients on treatment and at EOT (Fig. 5B, C). In PADA-1, acquired *ESR1* mutations were monitored through a sampling of ctDNA levels; this monitoring allowed switching of ET partners that prolonged PFS in patients treated with palbociclib plus ET [29]. Switching to fulvestrant provided a 6.2-month PFS advantage over continued treatment with an aromatase inhibitor in patients who developed *ESR1* mutations while on palbociclib and an aromatase inhibitor [30]. Oral selective estrogen receptor degraders such as elacestrant are a treatment option for patients who acquire *ESR1* mutations [31, 32]. These data illustrate the potential of early longitudinal ctDNA measurement for optimizing treatment plans.

The use of less-invasive liquid biopsy to collect longitudinal ctDNA samples was an advantage of this analysis; however, this technology and this analysis have some limitations. Detection of copy numbers in ctDNA samples is limited, especially for deletions; therefore, the data presented may underestimate the total frequency of amplified genes, large deletions, and rearrangements. Variability in ctDNA levels between patients could affect the VAF of mutations in individual genes (for example, see Fig. 5D, in which ctDNA was not detected at any time point, possibly due to the limitations of the assay). To avoid bias based on ctDNA levels, bTMB analysis was restricted to samples with detectable ctDNA (ctDNA fraction > 0 after PureCN estimation coupled with machine-learning refinement). As with commercially available assays and in most clinical trials, germline sequencing was not performed; germline mutations were removed through bioinformatics methods (see the “Methods” section). Additionally, our analysis was exploratory, and sample sizes were relatively small; further validation in larger cohorts will be required to establish the potential utility of longitudinal ctDNA analysis in guiding treatment.

## Conclusion

Ribociclib plus ET decreased ctDNA levels in Asian patients who responded to treatment with HR + /HER2 − ABC, regardless of starting dose. ctDNA levels at baseline correlated with PFS, and ctDNA increases during treatment allowed the identification of PD in a significant fraction of patients before it was detected by radiological methods. While this analysis is hypothesis generating, the results demonstrate the potential of measuring ctDNA throughout treatment to detect tumor progression, as well as of identifying genetic alterations that have clinical implications in HR + /HER2 − ABC.

### Supplementary Information

**Additional file 1.** Panel of Genes Used for cfDNA Analysis.

**Additional file 2:** **Supplementary Table 1.** cfDNA Samples Included in 23This Analysis^a^. Abbreviations: C5D1, cycle 5 day 1; cfDNA, cell-free DNA; EOT, end of treatment; max, maximum; min, minimum; pt, patient. ^a^One patient experienced failed quality control. ^b^Japanese group. ^c^Japanese and Asian non-Japanese groups. ^d^Asian non-Japanese group. **Supplementary Table 2.** Correlation Between ctDNA Fraction and Percentage Tumor Change between C5D1 and EOT^a,b^. Abbreviations: BL, baseline; C5D1, cycle 5 day 1; ctDNA, circulating tumor DNA; EOT, end of trial; PD, progressive disease; PR, partial response; pt, patient; SD, stable disease. ^a^Detected ctDNA was defined as ctDNA > 0. ^b^Patients with nondetectable ctDNA at baseline: 4 had PR/SD, 4 had PR->PD, and 2 had SD->PD. **Supplementary Figure 1.** Spider Plots and ctDNA Fraction of Individual Patients^a^ With (A) Progressive Disease, (B) Partial Response, or (C) Stable Disease. Abbreviations: AA, amino acid; ctDNA, circulating tumor DNA; EOT, end of treatment; frac, fraction. ^a^Patient number is not representative of a patient identifier.

## Data Availability

Novartis made the study protocols available for MONALEESASIA at the time of primary publications. Individual participant data will not be made available.

## Abbreviations

ABC: Advanced breast cancer
AE: Adverse event
BOR: Best overall response
bTMB: Tumor mutational burden calculated from plasma samples
C: Cycle
CDK4/6i: Cyclin-dependent kinase 4/6 inhibitor
CR: Complete response
ctDNA: Circulating tumor DNA
EOT: End of treatment
ET: Endocrine therapy
HER2: Human epidermal growth factor receptor-2
HR: Hormone receptor
max: Maximum
met: Metastatic
min: Minimum
mut/Mb: Mutations per megabase
NA: Not available
ORR: Overall response rate
OS: Overall survival
PD: Progressive disease
PFS: Progression-free survival
PR: Partial response
pt: Patient
RECIST: Response Evaluation Criteria in Solid Tumors
SD: Stable disease
WOC: Withdrawal of consent
