# Supplementary material for: Potential value of ctDNA monitoring in metastatic HR + /HER2 − breast cancer: longitudinal ctDNA analysis in the phase Ib MONALEESASIA trial
Source: BMC Med. 2023 Aug 15;21:306. doi: 10.1186/s12916-023-03017-z (PMC10426213; doi:10.1186/s12916-023-03017-z)
Supplement: Supplementary file 2 — Additional file 2: Supplementary Table 1. cfDNA Samples Included in 23This Analysisa. Abbreviations: C5D1, cycle 5 day 1; cfDNA, cell-free DNA; EOT, end of treatment; max, maximum; min, minimum; pt, patient. aOne patient experienced failed quality control. bJapanese group. cJapanese and Asian non-Japanese groups. dAsian non-Japanese group. Supplementary Table 2. Correlation Between ctDNA Fraction and Percentage Tumor Change between C5D1 and EOTa,b. Abbreviations: BL, baseline; C5D1, cycle 5 day 1; ctDNA, circulating tumor DNA; EOT, end of trial; PD, progressive disease; PR, partial response; pt, patient; SD, stable disease. aDetected ctDNA was defined as ctDNA > 0. bPatients with nondetectable ctDNA at baseline: 4 had PR/SD, 4 had PR->PD, and 2 had SD->PD. Supplementary Figure 1. Spider Plots and ctDNA Fraction of Individual Patientsa With (A) Progressive Disease, (B) Partial Response, or (C) Stable Disease. Abbreviations: AA, amino acid; ctDNA, circulating tumor DNA; EOT, end of treatment; frac, fraction. aPatient number is not representative of a patient identifier. [file 12916_2023_3017_MOESM2_ESM.docx]

**Supplementary Table 1. cfDNA Samples Included in This Analysis^a^**

| **Treatment** | **Pts,**  **n** | **Samples,**  **n** | **No. of on-treatment sample collection time points, n** | | | **C5D1 samples,**  **n** | **EOT samples,**  **n** |
| --- | --- | --- | --- | --- | --- | --- | --- |
|  |  |  | **Median** | **Min** | **Max** |  |  |
| **Ribociclib (300 mg) + letrozole^b^** | 6 | 48 | 9.5 | 3 | 12 | 6 | 6 |
| **Ribociclib (400 mg) + letrozole^c^** | 12 | 84 | 5 | 2 | 19 | 9 | 10 |
| **Ribociclib (600 mg) + letrozole^d^** | 7 | 84 | 16 | 2 | 18 | 7 | 2 |
| **Ribociclib (300 mg) + fulvestrant^b^** | 16 | 63 | 3.5 | 2 | 9 | 13 | 15 |
| **Ribociclib (300 mg) + letrozole^b^** | 15 | 118 | 7 | 1 | 15 | 13 | 6 |
| **Ribociclib (300 mg) + tamoxifen + goserelin^b^** | 15 | 102 | 5 | 2 | 14 | 15 | 11 |
| **Ribociclib (600 mg) + letrozole^d^** | 16 | 75 | 3.5 | 1 | 14 | 13 | 7 |
| **Total** | 87 | 574 |  |  |  | 76 | 57 |

Abbreviations: C5D1, cycle 5 day 1; cfDNA, cell-free DNA; EOT, end of treatment; max, maximum; min, minimum; pt, patient.

^a^ One patient experienced failed quality control.

^b^ Japanese group.

^c^ Japanese and Asian non-Japanese groups.

^d^ Asian non-Japanese group.

**Supplementary Table 2. Correlation Between ctDNA Fraction and the Percentage of Tumor Change Between C5D1 and EOT^a,b^**

|  | **Total**  **pts** | **Pts with  <30% reduction in tumor diameter at EOT , n** | **Pts with  >30 % reduction in tumor diameter at EOT, n** | **Pts with no change  in percentage of ctDNA from C5D1 to EOT, n ^b^** | **Pts with increase in  percentage of ctDNA from C5D1 to EOT, n ^b^** |
| --- | --- | --- | --- | --- | --- |
| **PR/SD** | 10 | 8 | 2 | 10 | 0 |
| **PR->PD** | 20 | 14 | 6 | 7 | 12 |
| **SD->PD** | 17 | 3 | 14 | 5 | 9 |

Abbreviations: BL, baseline; C5D1, cycle 5 day 1; EOT, end of trial; PD, progressive disease; PR, partial response; pt, patient; SD, stable disease.

^a^ Detected ctDNA is ctDNA >0.

^b^ Patients with nondetectable ctDNA at baseline: 4 with PR/SD; 4 with PR then PD, 2 with SD then PD.

**Supplementary Figure 1: Spider Plots and ctDNA Fraction of Individual Patients^a^ With (A) Progressive Disease, (B) Partial Response, or (C) Stable Disease**


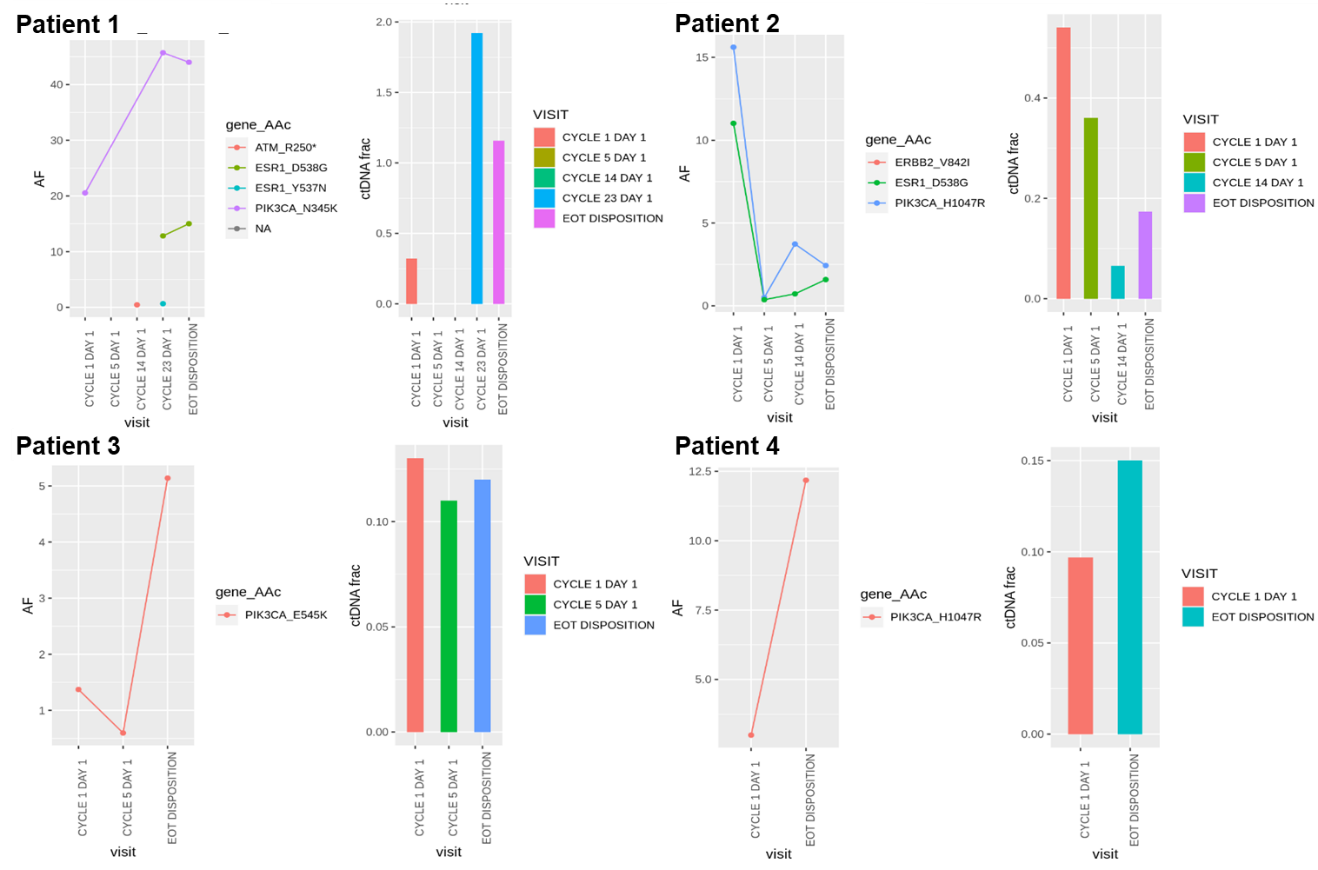
A. Patients with progressive disease as best response.

B. Patients with partial response as best response followed by disease progression.


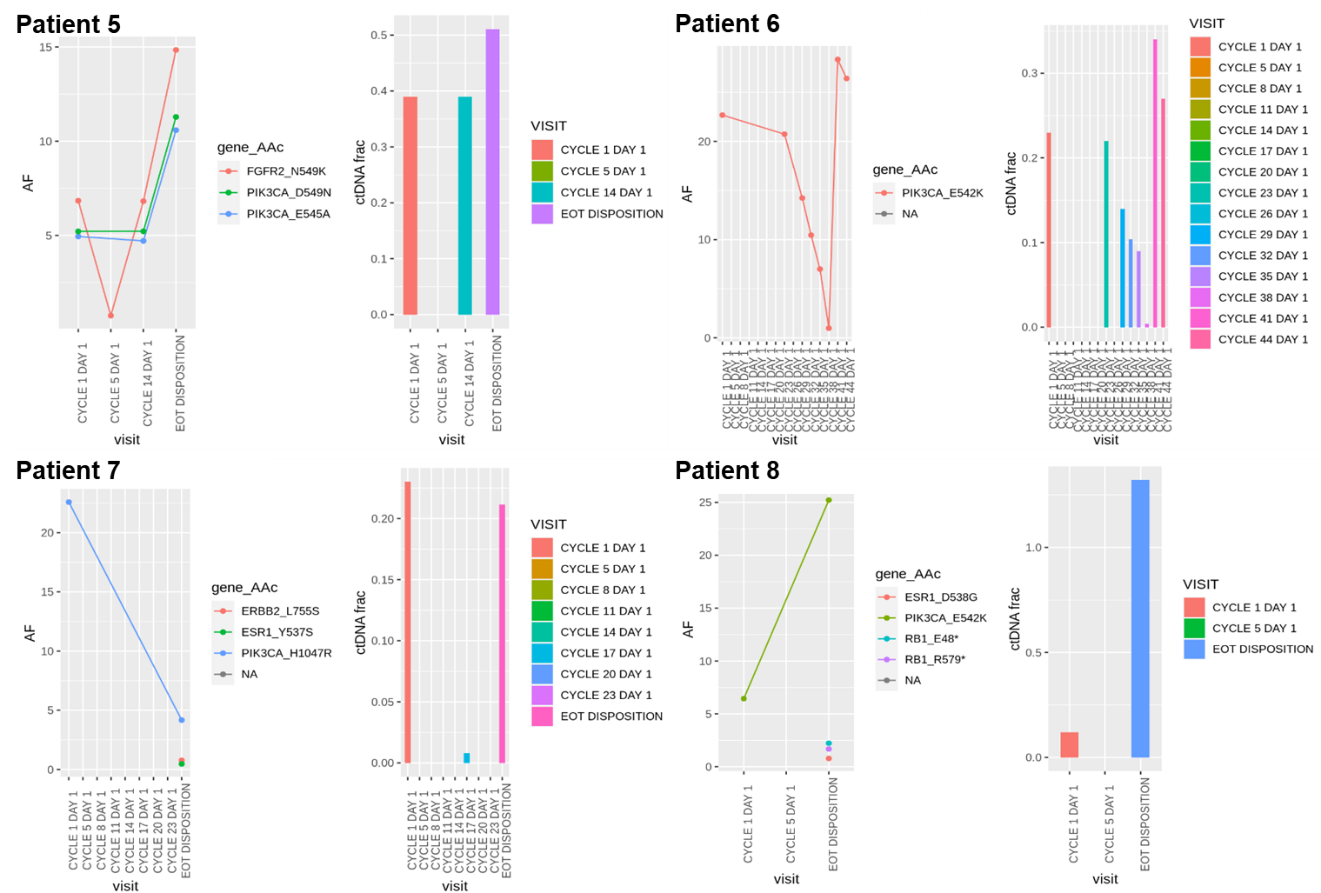


C. Patients with stable disease as best response followed by disease progression.
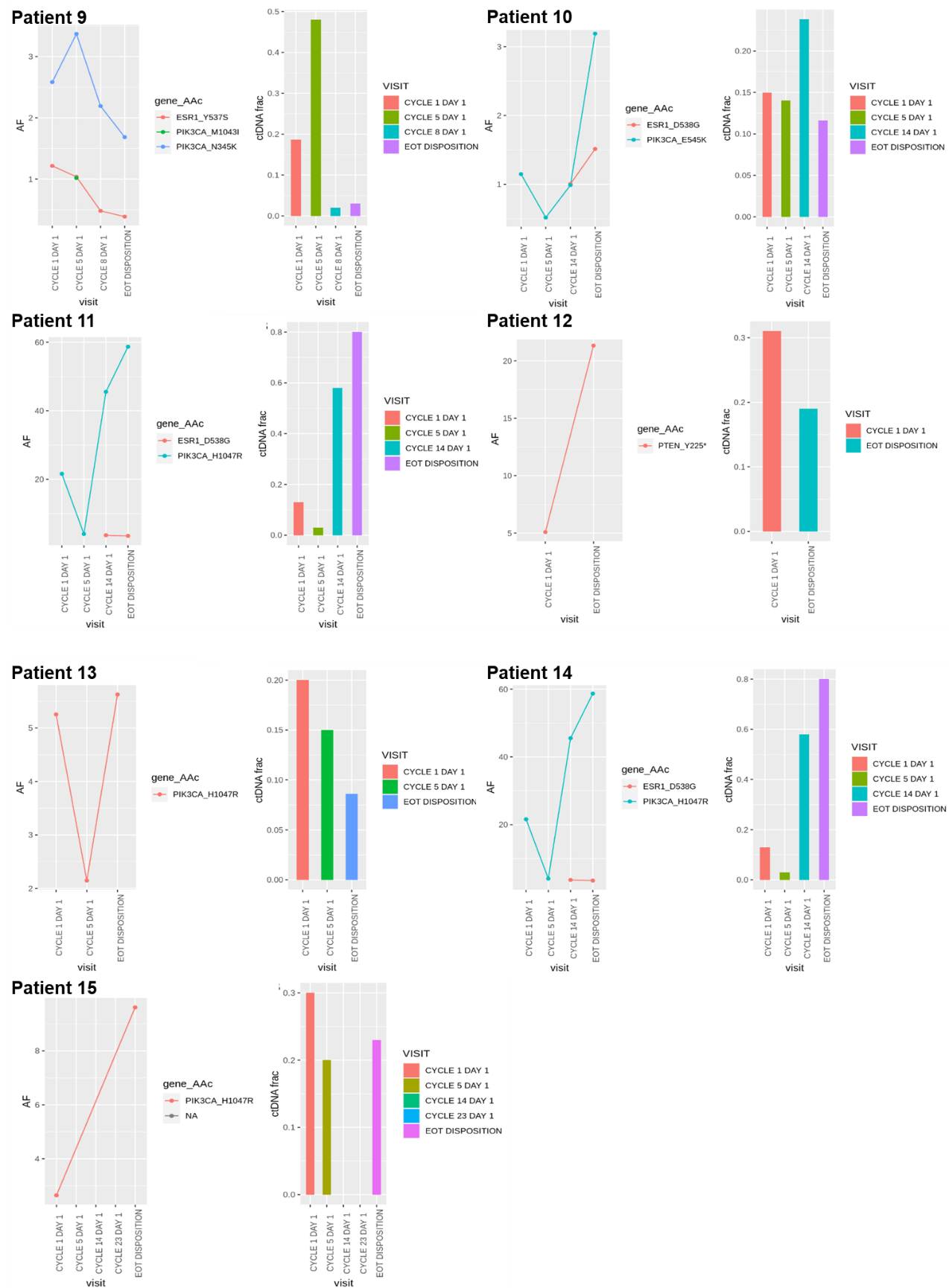


Abbreviations: AA, amino acid; ctDNA, circulating tumor DNA; EOT, end of treatment; frac, fraction.

^a^ Patient number is not representative of a patient identifier.
